# Reply to Letter to the Editor: “The added benefit of contrast-enhanced CT in the evaluation of incidental FDG-avid colon lesions”

**DOI:** 10.1007/s00259-020-04749-3

**Published:** 2020-06-30

**Authors:** Julian Kirchner, Benedikt M. Schaarschmidt, Firas Kour, Lino M. Sawicki, Ole Martin, Johannes Bode, Stephan vom Dahl, Verena Keitel, Dieter Häussinger, Christina Antke, Christian Buchbender, Gerald Antoch, Philipp Heusch

**Affiliations:** 1 Department of Diagnostic and Interventional Radiology, Medical Faculty, University Dusseldorf, Moorenstrasse 5, D-40225 Düsseldorf, Germany; 2 Clinic for Gastroenterology, Hepatology and Infectious Diseases, University Hospital Düsseldorf, Medical Faculty, Heinrich-Heine-University, Moorenstrasse 5, 40225 Düsseldorf, Germany; 3 Medical Faculty, Department of Nuclear Medicine, University Dusseldorf, 40225 Düsseldorf, Germany

We would like to thank Dr. Lee and colleagues for their well-thought-out questions and their interest in our work about the added benefit of contrast-enhanced CT in the evaluation of incidental FDG-avid colonic lesions [1].

First, while an overall incidence of focal ^18^F-FDG uptake in the colon has been observed in 1.3–3% [2–7] in the studies mentioned by Dr. Lee and colleagues, focal tracer uptake was detected in 38.4% in our cohort. Taking a closer look at the results, this difference is probably due to a different reference standard. All studies mentioned by Dr. Lee and colleagues calculated the incidence based on the total sum of all PET/CT examinations performed. Our study only included patients who had undergone contrast-enhanced ^18^F-FDG PET/CT and colonoscopy within 6 months after PET/CT. Compared with a non-selected patient population, the population in our study can, thus, be expected to have a higher incidence of colon pathology. This issue has been addressed in *Discussion*. Assessment for focal colonic ^18^F-FDG uptake was performed based on only the PET dataset; thus, the CT component had no influence on the incidence.

Second, in our study, the positive predictive value (PPV) was increased from 29 to 50% by including CT findings (wall-thickening, intraluminal nodule, and contrast enhancement). To our opinion, similar PPVs in the studies cited by Dr. Lee and colleagues may also be explained by difference in patient populations. In the study by Gutman et al., the PPV was 67%, but was calculated for all coloscopic abnormalities and included adenomas with mild to moderate dysplasia [4]. In the study by Lee et al., every type of adenoma was defined as pre-malignant [7], while in our study, adenomas without or with low-grade intraepithelial neoplasias were excluded, as they do not have clinical impact. The PPV given in the study of Israel et al. (46%) was calculated for the whole GIT; results for findings only in the colon are not given in this paper [5]. The highest PPV was reported by Even-Sapir et al. However, this study design aimed at the evaluation of malignant colonic tumours and pre-malignant lesions rather than all incidentally found lesions [3]. Only proven malignancies were included in the final analysis and even FDG-PET-negative masses detected on CT.
